# From the Cluttered Room to the Full Hard Drive: The Relationship Between Hoarding Disorder and Digital Hoarding

**DOI:** 10.3390/bs16030429

**Published:** 2026-03-16

**Authors:** Natalia Bravo-Adasme, Alejandro Cataldo, Elizabeth E. Grandón, Javiera Riquelme, Yasmin Rayo, Constanza Reyes

**Affiliations:** 1 Doctorado en Economía y Gestión de la Información, Facultad de Ciencias Empresariales, Universidad del Bío-Bío, Concepción 4030000, Chile; egrandon@ubiobio.cl; 2 Escuela de Ingeniería Informática Empresarial, Facultad de Economía y Negocios, Universidad de Talca, Talca 3460000, Chile; acataldo@utalca.cl (A.C.); jriquelme19@alumnos.utalca.cl (J.R.); yrayo18@alumnos.utalca.cl (Y.R.); coreyes@utalca.cl (C.R.)

**Keywords:** digital hoarding, hoarding disorder, stress, memory, enjoyment

## Abstract

The rapid expansion of digital information has given rise to new forms of hoarding that share features with hoarding disorder, yet their underlying psychological mechanisms remain insufficiently understood. Although hoarding disorder (HD) has been extensively studied, digital hoarding (DH) has received far less empirical attention within the behavioral sciences. This study examines whether the factors associated with hoarding disorder also account for digital hoarding and whether hoarding disorder influences digital hoarding. Using partial least squares structural equation modeling (PLS-SEM), data from 344 university students were analyzed, considering cognitive factors (memory, attention, and decision making) and emotional factors (stress, enjoyment, and anxiety). The results reveal that stress is the most influential factor in both behaviors. However, the relative importance of secondary factors differs: enjoyment plays a more prominent role in hoarding disorder, whereas memory is more salient in digital hoarding. Furthermore, hoarding disorder directly predicts digital hoarding and mediates the effects of stress, enjoyment, and memory, explaining 37.6% of the variance in digital hoarding. These findings suggest that digital hoarding is not merely an extension of hoarding disorder but rather a behavior characterized by distinct psychological dynamics.

## 1. Introduction

In recent decades, behaviors associated with the acquisition, storage, and accumulation of objects have undergone significant changes in both physical and digital realms. Globally, studies indicate that between 1.75 and 3.6% of the population display clinically significant hoarding behaviors (Postlethwaite et al., 2019), equating to 1 in 40 individuals affected by hoarding disorders (Mueller, 2025). Concurrently, in the digital context, a new form of hoarding has emerged as a parallel phenomenon, characterized by the constant and massive storage of various digital objects. Reports indicate that, on average, individuals have approximately 1000 unread emails, dozens of applications, many open browser tabs, and thousands of unorganized digital objects. Furthermore, 77% of these individuals report perceiving negative impacts from these behaviors (Koebert & Lane, 2025). Recent research has further documented the diverse contexts in which digital hoarding manifests, from social media platforms (Wu et al., 2024; Zhang et al., 2025) to professional organizational environments (McKellar et al., 2024), underscoring the pervasiveness of this phenomenon across multiple domains of digital life.

The magnitude of these data raises questions about people’s capacity to effectively organize and manage not only physical but also digital objects. From a physical perspective, this limitation has manifested in excessive hoarding behaviors, formally recognized in the Diagnostic and Statistical Manual of Mental Disorders (DSM-5) (American Psychiatric Association, 2013). In contrast, digital hoarding has received far less scholarly attention despite its potential impact on individuals’ well-being. Unlike tangible objects, the contemporary digital context fosters such behaviors due to low storage costs, virtually unlimited capacity, and a lack of tangible cues that reflect such accumulation (Neave et al., 2019; Sedera et al., 2022; Vinoi et al., 2024). More recent studies identified emotional attachment and disposal barriers as antecedents of hoarding behavior among Generation Z users (Zhang et al., 2025). In workplace contexts, authors have documented that organizational culture and environmental pressures amplify digital hoarding tendencies, suggesting that this phenomenon is not limited to personal settings but extends to professional environments, with specific implications for data governance and cybersecurity (McKellar et al., 2024).

From a psychological and behavioral perspective, hoarding disorder has been extensively studied, yielding a cognitive–behavioral model that proposes interactions among deficits in cognitive processes (such as memory and attention), dysfunctional beliefs, and emotional responses as the primary mechanisms generating and sustaining hoarding behaviors (Frost & Hartl, 1996; Steketee & Frost, 2007). However, research on digital hoarding, though burgeoning, remains incipient. Studies have proposed some theoretical explanations for the causes of this behavior, yet few have delved into it in greater depth (Mckellar et al., 2020; Sillence et al., 2023; Sweeten et al., 2018).

Additionally, extant research has analyzed the correlations between both behaviors, establishing that individuals struggling to manage their physical objects also experience similar problems in digital domains (Luxon et al., 2019; Thorpe et al., 2019). However, these analyses have focused solely on establishing correlations without exploring the underlying causes that may explain the potential transfer of behavior between physical and digital contexts. This constitutes a critical gap in the literature: while the co-occurrence of hoarding disorder and digital hoarding has been documented, the mechanistic pathways through which hoarding disorder symptoms may influence digital hoarding behavior remain unexplored. Specifically, it is unclear whether individuals with hoarding disorder exhibit higher levels of digital hoarding not merely because of behavioral overlap, but because the cognitive and emotional processes central to hoarding disorder symptoms—such as impaired memory confidence, attentional biases, decision-making difficulties, and heightened emotional reactivity—directly drive digital hoarding behaviors.

Based on this, the present study examines the cognitive and emotional factors underlying both hoarding disorder (HD) and digital hoarding (DH). Specifically, it addresses two research questions: (1) do the factors influencing hoarding disorder overlap with those driving digital hoarding? and (2) does hoarding disorder predict digital hoarding? To this end, three cognitive factors (memory, attention, and decision-making) and three emotional factors (enjoyment, stress, and anxiety) were analyzed and evaluated for their effects on both behaviors. A structural equation modeling approach (PLS-SEM) was applied in a sample of 344 university students. The present study is grounded in the cognitive–behavioral model (Frost & Hartl, 1996; Steketee & Frost, 2007), extending it to the digital domain to examine whether the same cognitive (memory, attention, and decision-making) and emotional (enjoyment, stress, and anxiety) factors that underlie hoarding disorder symptoms are also implicated in digital hoarding. This theoretical extension is motivated by the documented behavioral parallels between both phenomena; yet, to date, no study has systematically tested whether the cognitive–behavioral profile of hoarding disorder applies to digital hoarding.

The theoretical contribution of this study is twofold. First, it provides a systematic test of whether the cognitive–behavioral model of hoarding disorder—which has been validated in hoarding disorder research—also applies to digital hoarding, identifying which specific cognitive and emotional factors emerge as shared or domain-specific antecedents. Second, it advances the literature by modeling hoarding disorder symptoms as a direct predictor of digital hoarding, thereby examining whether hoarding disorder tendencies transfer to the digital domain beyond the influence of common cognitive and emotional factors.

This article is structured into five main sections. The following section summarizes key studies on hoarding disorder and digital hoarding to develop the research hypotheses. The third section outlines the methodology applied in this study. The fourth section reports the main results. The fifth section discusses these findings, and the final section offers conclusions along with avenues for future research.

## 2. Theoretical Framework

### 2.1. Hoarding Disorder (HD)

Hoarding Disorder was initially defined as the acquisition of excessive possessions and a persistent failure to discard them despite their lack of apparent value or utility for most individuals (Frost & Gross, 1993). The literature identifies three key components of this behavior: the excessive acquisition of objects, pronounced difficulty discarding them, and accumulation of clutter that interferes with the functional use of different living spaces (Frost & Hartl, 1996). Early research on hoarding behavior revealed that affected individuals experience significant distress when confronted with discarding possessions, often ascribing sentimental, instrumental, or even aesthetic value (Frost et al., 2004; Frost & Hartl, 1996).

This behavior was formally recognized as hoarding disorder in 2013 by the DSM-5 (American Psychiatric Association, 2013). Previously regarded solely as a symptom of obsessive–compulsive disorder (OCD), it was reclassified based on various patterns, clinical studies, and treatment evidence (Mataix-Cols et al., 2010). According to the DSM-5, hoarding disorder arises when an individual presents persistent difficulty discarding or parting with possessions objects, driven by a perceived need to preserve them and associated distress upon disposal. This leads to the excessive accumulation of objects, resulting in clutter that significantly impairs the functional use of living spaces (American Psychiatric Association, 2013).

The effects of hoarding disorder are well-documented in the literature. Affected individuals experience significant impairments in occupational functioning, physical health, and interpersonal and family relationships, often rendering their living spaces inaccessible and unsafe (Büscher et al., 2014; Nutley et al., 2022; Sekhon & Leontieva, 2023; Tolin, 2023; Tolin et al., 2008). In student populations, research has linked hoarding disorder to impaired social relationships, elevated anxiety and depression, difficulties in managing personal space, and diminished academic performance (Coles et al., 2003; Dozier et al., 2025; Tolin et al., 2019).

### 2.2. Digital Hoarding (DH)

The advent of technology and the widespread use of digital devices have given rise to a new manifestation of acquisition and storage behavior known as digital hoarding. This phenomenon has been defined as the constant acquisition of digital content coupled with an inability to discard or manage it effectively, resulting in digital clutter (Sedera et al., 2022; Sedera & Lokuge, 2018). Unlike physical possessions, which occupy tangible spaces and yield visible consequences upon excessive accumulation, digital items can be stored almost indefinitely due to abundant low-cost virtual space capacity, thereby enabling accumulation without obvious visual cues.

Digital hoarding manifests in different forms: thousands of unread stored emails, repeatedly downloaded files or documents scattered across disorganized folders, thousands of duplicate photographs or videos across multiple electronic devices, hundreds of open browser tabs, or unused downloaded applications. Each of these reflects a pattern akin to hoarding disorder, characterized by compulsive acquisition of digital items and persistent difficulty discarding them.

As previously mentioned, a defining characteristic of the digital context is its expansive storage capacity, spanning physical devices (e.g., USB drives, hard drives, computers) and virtual repositories or cloud services (Sedera et al., 2022; Sweeten et al., 2018). Unlike hoarding disorder, where physical storage space is inherently limited; digital hoarding can proliferate almost indefinitely and with subtler evident consequences. Current studies link digital hoarding to elevated stress, anxiety, and reduced productivity (McKellar et al., 2024; Sedera et al., 2022; Sedera & Lokuge, 2018), while research on student populations indicates associations with increased mental fatigue, cognitive errors, and decreased academic performance (Bravo-Adasme et al., 2025; Liu & Liu, 2025).

### 2.3. Factors Influencing Hoarding Disorder and Digital Hoarding

The cognitive-behavioral model provides one of the most widely used frameworks for understanding the causes of hoarding (Frost & Hartl, 1996). It posits that various cognitive and emotional processes interact to cause and maintain hoarding behaviors. Specifically, it proposes that hoarding behaviors such as clutter, acquisition, and retention arise from vulnerability factors rooted in past experiences. These factors engender maladaptive beliefs and attachments, which in turn elicit both positive and negative emotional reactions that ultimately lead individuals to develop hoarding disorder (Steketee & Frost, 2007).

Among the model’s central components are deficits in information-processing such as memory, attention, categorization, and decision-making, alongside maladaptive beliefs about possessions and intense emotional responses, both positive and negative, to acquisition and discarding (Steketee et al., 2003). Several empirical studies have validated the components of this model. For example, authors have demonstrated that deficits in executive functions such as memory and decision-making significantly predict the severity of hoarding (Frost et al., 2011; Tolin et al., 2012). Additionally, studies have shown that a higher incidence of stressful events preceding or coinciding with hoarding generated stronger object attachment (Fontenelle et al., 2021). Likewise, positive correlations have been identified between anxiety and stress with higher hoarding severity (Tolin et al., 2011).

Research on the causes of digital hoarding remains nascent and, therefore, limited in scope. Existing literature identifies some causes that lead people to hoard digital objects, including regret, materialism, fear of missing out (FoMo), attachment, among others (Luxon et al., 2019; Mckellar et al., 2020; Sweeten et al., 2018; Van Bennekom et al., 2015; Vinoi et al., 2024). However, some of these factors lack empirical validation, relying predominantly on theoretical propositions.

### 2.4. Theoretical Gap and Hypothesis Development

Despite the growing emergence of digital hoarding as a new form of hoarding, significant gaps persist in understanding its underlying predictors. Extant literature on hoarding disorder provides robust evidence for its multiple cognitive and emotional causes (Büscher et al., 2014; Frost & Hartl, 1996; Steketee & Frost, 2007; Tolin, 2023, p. 202; Tolin et al., 2011). However, research on digital hoarding remains in early stages, with very few empirical investigations examining whether these causal mechanisms extend to the digital domain.

To date, no empirical studies have evaluated whether the causes identified in the model proposed by Frost and Hartl (1996) such as memory, attention, or decision-making, predict digital hoarding to the same extent as hoarding disorder, nor whether their relative salience differs across physical and digital contexts, given the distinct affordances that characterize digital environments. Likewise, although emotional reactions such as enjoyment, anxiety, and stress have been extensively assessed in hoarding disorder, their role in digital hoarding remains unexplored. This dual gap—concerning both the cognitive and emotional antecedents of digital hoarding, and the direct predictive relationship between hoarding disorder and digital hoarding as a behavioral outcome—constitutes the central motivation of the present study.

#### 2.4.1. Anxiety and Its Influence on Hoarding Disorder Symptoms and Digital Hoarding

Anxiety constitutes a future-oriented affective state wherein individuals anticipate and prepare to cope with upcoming negative events (Nathan & Gorman, 2002). The literature has established a consistent relationship between anxiety and hoarding disorder, whereby individuals with HD exhibit elevated rates of comorbidity with anxiety disorders (Frost et al., 2011; Timpano et al., 2013). This relationship is grounded in the hoarding’s roles in emotional regulation, which is associated with temporary alleviation of anxiety related to loss or discarding of possessions (Steketee et al., 2003).

Anxiety may also underpin digital hoarding. Although no empirical research have directly linking the two constructs, authors have determined that anxiety is one of the dimensions associated with the development of digital hoarding, irrespective of digital files’ (e.g., emails) actual value or management (Mckellar et al., 2020). Similarly, Luxon et al. (2019) reported anxiety when individuals were required to delete certain photographs on Pinterest. Based on the above, the following hypotheses are proposed.

**H1.**  *Anxiety influences hoarding disorder.*

**H7.**  *Anxiety influences digital hoarding.*

#### 2.4.2. Stress and Its Influence on Hoarding Disorder Symptoms and Digital Hoarding

Stress is defined as the perception of threat, eliciting consequent anxiety, discomfort, emotional tension, and impaired adaptability (Fink, 2016). Authors suggest that stress may be a contributing factor to hoarding disorder. In some cases, hoarding may confer a sense of security or control amid stressful or uncertain circumstances (Frost et al., 2004). Additionally, another study links stress, particularly interpersonal stress, is associated with significantly more severe hoarding symptoms (Timpano et al., 2011).

Negative emotions associated with hoarding symptoms, such as stress, may also manifest in digital contexts. Although no empirical research has directly linked these constructs, some authors have suggested that stress is one of the dimensions driving digital hoarding behaviors, particularly amid information overload (Neave et al., 2019). Furthermore, other authors mention that participants in their study reported experiencing significant stress associated with managing accumulated digital files, suggesting a bidirectional relationship wherein stress both precipitates and arises from digital (Sweeten et al., 2018). Based on the above, the following hypotheses for this research are proposed.

**H2.**  *Stress influences hoarding disorder.*

**H8.**  *Stress influences digital hoarding.*

#### 2.4.3. Enjoyment and Its Influence on Hoarding Disorder Symptoms and Digital Hoarding

Enjoyment is defined as the degree to which individuals experience joy, happiness, and satisfaction in a given situation (Menon & Kahn, 2002). Positive emotions such as enjoyment are evoked by thoughts of the sentimental, instrumental, and aesthetic value of possessions (Frost et al., 1998; Frost & Hartl, 1996). Contrary to common assumptions about hoarders, research indicates that individuals with hoarding disorders experience certain levels of enjoyment from their possessions (Grisham & Barlow, 2005). This aligns with Mataix-Cols et al. (2010) who found that individuals with hoarding disorder associate saving and acquisition with positive emotions such as excitement, enjoyment, and euphoria.

Enjoyment may also be associated with digital hoarding. Downloading or saving digital objects may be associated with immediate satisfaction and a sense of security, particularly when perceived as relevant. Although no studies directly demonstrate enjoyment’s influence on digital hoarding, research indicates that individuals who hoard for collection purposes expressed enjoyment from saving and organizing digital objects with clearly attributed value (Mckellar et al., 2020). Based on the above, the following hypotheses are proposed.

**H3.**  *Enjoyment influences hoarding disorder.*

**H9.**  *Enjoyment influences digital hoarding.*

#### 2.4.4. Attention and Its Influence on Hoarding Disorder Symptoms and Digital Hoarding

Attention is defined as the set of cognitive operations that select and sustain conscious events (Baars, 1997). Research indicates that individuals exhibiting high levels of hoarding simultaneously display attention deficits that foster clutter, a hallmark characteristic of the disorder (Hartl et al., 2004; Tolin et al., 2011). Similarly, Grisham et al. (2007) found that hoarding patients struggled to identify targets, exhibiting impaired spatial attention alongside slower and more variable reaction times. These attentional difficulties contribute to hoarding by impeding sustained focus when organizing or discarding items, while fostering proneness to distraction.

In the digital environment, attention is constantly fragmented by persistent notifications across multiple devices and concurrent applications use, which may reduce attention and influence storage behaviors and habits. Consequently, individuals with attentional deficits may struggle to sustain focus when organizing their digital content. This arises from their proneness to distraction, which hinders evaluation of the relevance of stored digital objects and reinforcing indiscriminate saving “just in case” (Sweeten et al., 2018). Based on the above, the following hypotheses are proposed.

**H4.**  *Attention influences hoarding disorder.*

**H10.**  *Attention influences digital hoarding.*

#### 2.4.5. Decision-Making and Its Influence on Hoarding Disorder Symptoms and Digital Hoarding

Decision-making constitutes a fundamental human cognitive process whereby individuals select an option or course of action from the available alternatives based on personal criteria (Wang & Ruhe, 2007). This domain has been extensively studied in hoarding disorders, with research revealing pronounced decision-making impairments among affected individuals compared to those who do not suffer from the disorder, evidencing deterioration in this cognitive function (Dozier & Ayers, 2017; Kyrios et al., 2018; Tolin et al., 2012). Problems in decision-making are associated with hoarding, as indecision regarding objects disposal inevitably fosters progressive accumulation.

The decision-making difficulties associated with hoarding symptoms may also be related to digital hoarding. In digital contexts, individuals confront myriad choices regarding the retention or deletion of digital objects, often inducing decision fatigue. This may prompt individuals to indiscriminate storage over deliberate evaluation of relevance or utility of digital objects, thereby contributing to digital clutter accumulation (Zaremohzzabieh et al., 2024). Based on the above, the following hypotheses are proposed.

**H5.**  *Decision-making influences hoarding disorder.*

**H11.**  *Decision-making influences digital hoarding.*

#### 2.4.6. Memory and Its Influence on Hoarding Disorder Symptoms and Digital Hoarding

Memory is defined as the capacity to store and subsequently retrieve information (Zlotnik & Vansintjan, 2019). Studies on hoarding disorder identify memory deficits as a significant factor associated with this behavior. Authors have shown that patients with HD exhibit significantly poorer delayed verbal and visual recall compared with healthy individuals (Hartl et al., 2004). These memory deficits are associated with discarding difficulties, as patients fear forgetting important information contained in objects or losing access to valuable memories associated with them (Kyrios et al., 2018, p. 201; Nedeljkovic et al., 2009).

The memory deficits associated with HD may operate analogously in digital contexts. Individuals may develop attachment to digital data, particularly self-created tasks and projects (Sillence et al., 2023). This attachment stems from concerns about forgetting relevant information or losing valued memories, thereby prompting the accumulation of extensive digital objects. Based on the above, the following hypotheses are proposed.

**H6.**  *Memory influences hoarding disorder.*

**H12.**  *Memory influences digital hoarding.*

#### 2.4.7. Hoarding Disorder Symptoms and Its Influence on Digital Hoarding

Hoarding disorder may relate to digital hoarding. Although research directly linking both types of hoarding is scarce, recent studies have begun to identify patterns between these behaviors. These investigations reveal a significant correlation between HD and DH, with individuals struggling to discard physical objects exhibiting similar difficulties when managing their digital possessions, including files, emails, photographs, and applications (Luxon et al., 2019; Thorpe et al., 2019). Based on the above, the following hypothesis is proposed for this study.

**H13.**  *Hoarding disorder influences Digital Hoarding.*

**Figure 1:**
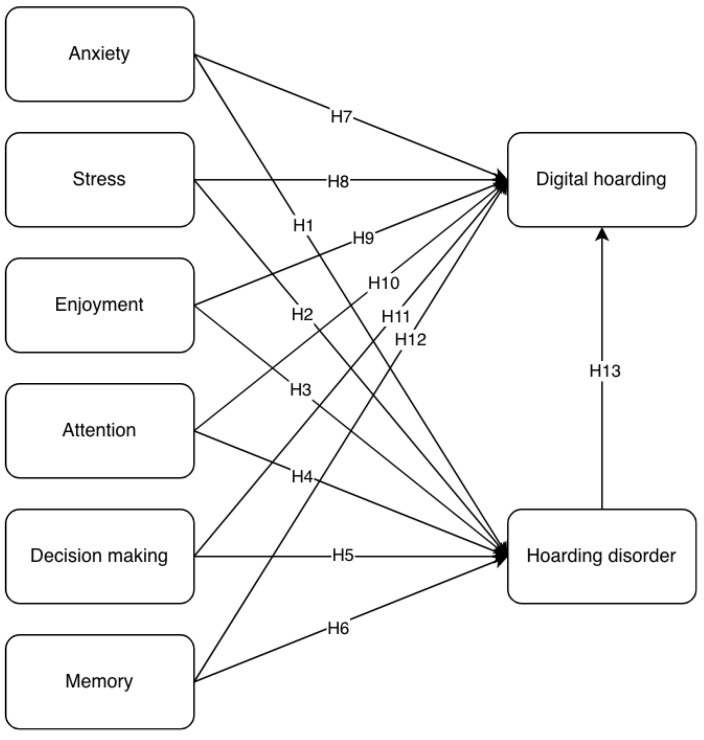
Proposed hypothesis model.

Figure 1 below presents the proposed hypothesis model.

## 3. Materials and Methods

Data collection for this study was conducted through the administration of a self-administered questionnaire applied to university students. The study was carried out at a Chilean university campus, and participants were recruited using a convenience sampling strategy (Etikan, 2016), which was deemed appropriate given the exploratory nature of the research and the accessibility of the target population. Data were collected in person, in paper-and-pencil format, in public spaces of the university campus, including study rooms, cafeterias, and rest areas. It should be acknowledged, however, that the use of convenience sampling limits the generalizability of the findings to broader populations. As participants were recruited from a single university campus in Chile, the sample may not be representative of other demographic groups or cultural contexts. Selection bias is an inherent limitation of this approach, given that individuals present in the surveyed spaces may differ systematically from those who were not reached. These constraints are discussed further in the limitations section of this article.

To be eligible for participation, individuals were required to be at least 18 years of age and to be actively enrolled in a degree program at the studied institution. These criteria were established to ensure participants had sufficient cognitive maturity to respond to the constructs assessed and to guarantee the relevance of the sample to the study population. Participation was voluntary and anonymous, and informed consent was obtained from all respondents prior to data collection. The study was conducted in accordance with the Declaration of Helsinki and received approval from the Scientific Ethics Committee of the university where the study was carried out.

The questionnaire was constructed using items adapted from and validated in previous research (Table 1). The complete questionnaire consisted of 79 items: 4 related to attention, 15 items to memory, 5 to decision-making, 3 to enjoyment, 7 to anxiety, 10 to stress, 5 to hoarding disorder, and 23 to digital hoarding. In addition, 7 demographic questions were included to characterize the participants. Finally, two control items were incorporated to ensure response consistency.

To ensure participants’ comprehension of the items, a pilot test was conducted with eleven university students. Minor revisions were made to clarify the meaning of some items based on pilot feedback; responses from this phase were excluded from the final analyzed sample. Following these adjustments, in-person data collection yielded 366 completed questionnaires. Of these, 22 were excluded for failing to meet the validation criteria, resulting in a final dataset of 344 questionnaires for analysis. Regarding data completeness, each questionnaire was manually reviewed for completeness by the research team at the moment of collection; incomplete responses were not retained in the dataset.

All instruments included in the questionnaire have established psychometric properties and prior use in student and non-clinical populations. The Memory and Cognitive Confidence Scale (MACCS) provided items for the memory, attention, and decision-making subscales. Exploratory factor analysis in a non-clinical student sample (n = 267) supported a four-factor structure, with Cronbach’s alpha coefficients ranging from α = 0.79 to 0.93 across subscales and α = 0.92 for the total scale (Nedeljkovic & Kyrios, 2007). The perceived enjoyment items were adapted from Sun and Zhang (2006), a widely used measure in technology acceptance research that has demonstrated adequate construct validity in university student samples. The anxiety subscale (7 items) was drawn from the Depression Anxiety Stress Scales (DASS-21), which demonstrated a well-defined three-factor structure, with a Cronbach’s alpha of α = 0.87 for the anxiety subscale specifically (Antony et al., 1998). Perceived stress was assessed using the Perceived Stress Scale, one of the most widely used instruments for measuring subjective stress appraisal, extensively validated in student and adult samples, demonstrating adequate internal consistency, acceptable reliability, and convergent validity with measures of psychological distress (Cohen & Williamson, 1988). Hoarding disorder was assessed using the Hoarding Rating Scale-Interview (Tolin et al., 2010), which showed high internal consistency (α = 0.97), strong cross-context reliability, and robust known-group validity, clearly differentiating individuals with hoarding disorder from OCD participants and non-clinical controls across all items. Finally, digital hoarding was measured using items adapted from the Saving Inventory-Revised (Frost et al., 2004), which presents a well-validated three-factor structure (difficulty discarding, clutter, and acquisition) and high internal consistency (α = 0.92 total; subscale alphas ranging from 0.87 to 0.91).

The methodological analysis comprised three phases. The first two phases were based on the disjoint approach, which is recently recommended for analyzing higher-order models due to its flexibility (Hair et al., 2024). The first phase entailed estimating the lower-order components (LOCs). Only the LOCs were modeled, omitting the higher-order construct (HOC). Latent scores for the LOCs were then calculated using conventional PLS-SEM, and internal consistency (α > 0.7), convergent validity (AVE > 0.5), and discriminant validity (HTMT < 0.85) were assessed per established guidelines (Hair et al., 2021, 2016).

In the second phase of the analysis, the higher-order construct was integrated. The LOCs were replaced by their latent scores, which then acted as indicators of the HOC. All exogenous variables retained their original indicators, preserving the relational structure of the theoretical model. Quality metrics (composite reliability and VIF for formative specifications) were re-evaluated considering the new configuration. At this final stage, the moderating effect of digital hoarding (DH) was analyzed.

The third phase consisted of conducting an endogeneity analysis using control variables. For this analysis, a PROCESS (Hayes, 2013) implemented in SmartPLS 4.0 software was employed. Three control variables were included: age, sex, and number of devices.

## 4. Results

Table 2 summarizes descriptive statistics for the participant sample, disaggregated by sex. Of the total sample, 54.36% identified as women, 44.18% as men, and 1.45% preferred not to disclose their gender. Age ranged from 18 to 44 years, with averages of 20.52 years for women and 20.38 for men. Participants also reported the number of electronic devices owned, ranging from zero to six, with medians of two devices for women and three devices for men.

Partial least squares structural equation modeling (PLS-SEM) was chosen because it is a non-parametric technique that does not impose strict multivariate normality assumptions on data distribution (Hair et al., 2022, 2024; Ravand & Baghaei, 2016). In contrast to covariance-based methods (CB-SEM), PLS-SEM relaxes the traditional assumptions of homoscedasticity and error independence by using resampling procedures such as bootstrapping to assess the statistical significance of parameters (Hair et al., 2022; Ravand & Baghaei, 2016).

The total sample comprised 344 cases, exceeding the two most widely accepted criteria for PLS-SEM models. Employing the inverse square root method (Kock & Hadaya, 2018), prospectively assuming a minimum path coefficient of 0.15, a 5% significance level, and 80% statistical power, the required minimum sample size is estimated at 275 cases. Additionally, based on the recommendations of Hair et al. (2021), 155 cases suffice for small path coefficients between 0.11 and 0.20, at 5% significance and 80% power. Therefore, the sample size substantially exceeds minimum requirements for analysis. To assess common method bias, Harman’s single-factor test was conducted, yielding 24.7% explained variance, well below the 50% threshold, indicating no such bias.

### 4.1. First-Stage Measurement Model Assessment

**Figure 2:**
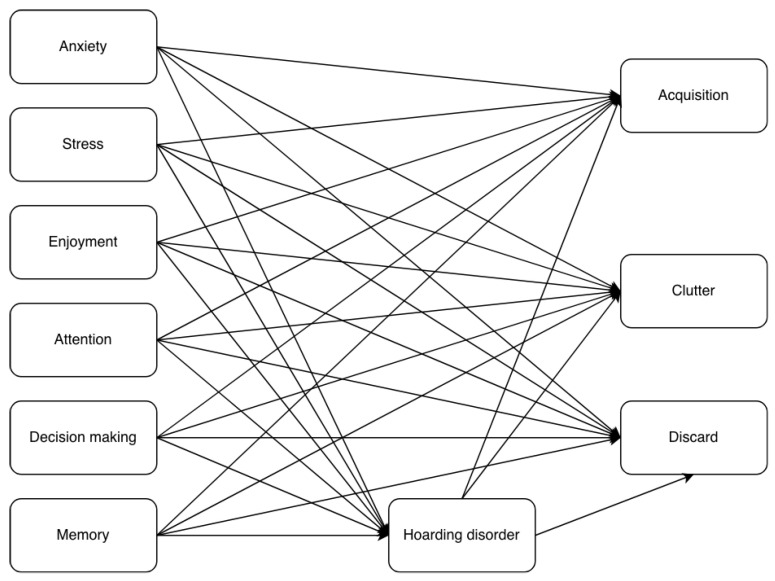
First-stage model.

The first-stage model included anxiety, stress, enjoyment, attention, decision-making, and memory as exogenous constructs, with hoarding disorder as the dependent variable (see Figure 1). In this model, difficulty discarding, clutter, and acquisition are dimensions or lower-order constructs of digital hoarding and were therefore included as independent endogenous variables. The higher-order construct digital hoarding was not included. Consistent with the structure of the original conceptual model (Figure 2), hoarding disorder mediates the relationships between anxiety, stress, enjoyment, attention, decision-making, and memory, and difficulty discarding, clutter, and acquisition.

In the first step of the assessment, item loadings were evaluated based on two selection criteria. Given the exploratory nature of the model, items with loadings greater than 0.65 were retained, a threshold higher than that typically required for this type of model (Hair et al., 2022), provided that no more than 20% of the total items were removed to prevent measurement issues. Although the model exhibited acceptable fit after removing six items, a total of 14 items were ultimately removed to ensure an adequate model fit. This corresponds to 19.44% of the total items, remaining below the 20% threshold.

Table A1 in Appendix A presents the final item loadings, which ranged from 0.674 to 0.914, indicating acceptable levels and supporting construct validity. Reliability, convergent validity, and discriminant validity were established. Table A2 reports Cronbach’s alpha, composite reliability, AVE, and HTMT values for each construct. Cronbach’s alpha values ranged from 0.787 to 0.952, and composite reliability (Rho_c) ranged from 0.857 to 0.958, both exceeding the minimum acceptable threshold of 0.70, thereby confirming good reliability. Convergent validity, assessed using AVE, ranged from 0.537 to 0.787, all above the minimum acceptable value of 0.50. Discriminant validity, assessed using HTMT, ranged from 0.074 to 0.824, remaining below the 0.85 threshold and indicating adequate construct distinctiveness. Finally, Table A1 reports VIF values ranging from 1.393 to 4.550, all below the maximum acceptable limit of 5.0, indicating no multicollinearity issues.

### 4.2. Second-Stage Measurement Model Assessment

**Figure 3:**
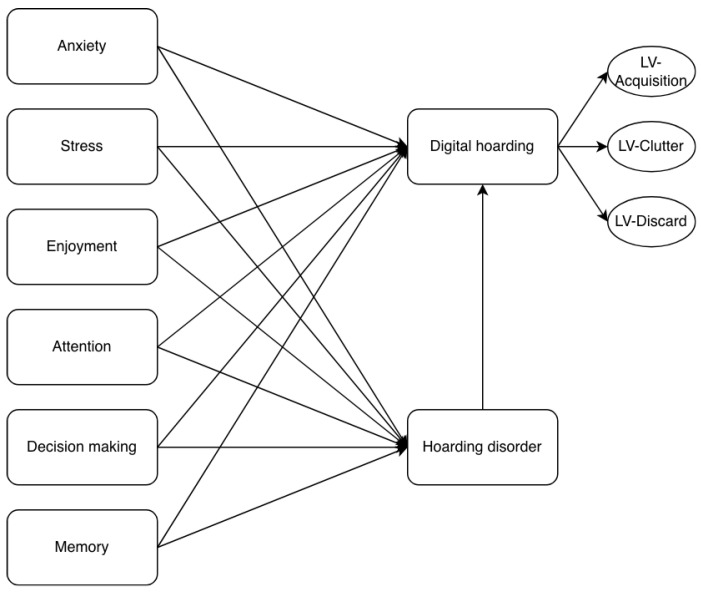
Second-stage model.

The second-stage model incorporated the same first-order constructs as the first-stage model (anxiety, stress, enjoyment, attention, decision-making, memory, and hoarding disorder). However, unlike the first-stage model, it also included the second-order construct of digital hoarding, while the lower-order constructs (difficulty discarding, clutter, and acquisition) modeled as indicators of digital hoarding using their latent variable scores (see Figure 3).

The measurement model assessment followed the same criteria for evaluating reliability, convergent validity, and discriminant validity as described above. Table A2 in Appendix A presents the final item loadings, which ranged from 0.711 to 0.914, all exceeding the acceptable thresholds and supporting construct validity. Table A3 reports Cronbach’s alpha, composite reliability, AVE, and HTMT values for each construct in the second-order model. Cronbach’s alpha values ranged from 0.808 to 0.952, and composite reliability (Rho_c) ranged from 0.869 to 0.958, both indicating strong reliability. AVE values ranged from 0.571 to 0.787, supporting convergent validity, while HTMT values ranged from 0.074 to 0.759, demonstrating adequate discriminant validity. Finally, Table A4 shows VIF values ranging from 1.390 to 4.550, confirming the absence of multicollinearity among the constructs.

### 4.3. Structural Model Evaluation

To test the relationships between the independent and dependent variables proposed in the conceptual model (Figure 1), the structural model was assessed. The analysis employed the bootstrapping procedure (10,000 samples, significance level of 5%, two-tailed). Table 3 presents the bootstrapping results for the model’s endogenous constructs.

The results indicate that the same factors significantly associations with both hoarding disorder symptoms and digital hoarding: enjoyment, memory, and stress. In contrast, anxiety, attention, and decision-making did not show significant effects on either hoarding disorder symptoms or digital hoarding. Moreover, the effects of the significant exogenous constructs on both hoarding disorder symptoms and digital hoarding were small in magnitude. Stress emerged as the most influential factor for both hoarding disorder symptoms and digital hoarding (β = 0.260 and β = 0.177, respectively). Interestingly, the second most influential factor for hoarding disorder symptoms was enjoyment, followed by memory (β = 0.140 versus β = 0.134), whereas for digital hoarding, the order was reversed, with memory preceding enjoyment (β = 0.150 versus β = 0.107).

The overlap between significant and non-significant exogenous variables reported in Table 3 suggests that the same factors influence both behaviors, thereby providing a positively answer to the first research question of this study. The direct effect of hoarding disorder on digital hoarding was positive and significant, exhibiting a large effect size (H13).

Next, the mediation effect of hoarding disorder symptoms on the relationship between each exogenous construct and digital hoarding was examined. Table 3 presents the path coefficients for the indirect and total effects of the model’s exogenous constructs (anxiety, attention, decision-making, enjoyment, memory, and stress) on digital hoarding. Once again, no significant effects were found for anxiety, attitude, or decision-making, confirming that these constructs did not influence digital hoarding.

As shown in Table 3 (direct effects) and Table 4 (indirect and total effects), the path coefficients for the direct, indirect, and total effects of enjoyment, memory, and stress on digital hoarding were all positive. These results indicate that the mediation of hoarding disorder symptoms on digital hoarding is of the complementary partial type. Once again, stress exerted the stronger influence on digital hoarding (β = 0.260), followed by memory (β = 0.195), and enjoyment (β = 0.154). In other words, hoarding disorder symptoms acted as a partial mediator that amplified the effects of enjoyment, memory, and stress on digital hoarding behavior.

Table 5 shows the coefficients of determination (R^2^) and the adjusted coefficient of determination (R^2^ adj) for hoarding disorder symptoms and digital hoarding. The structural model analysis indicates that the coefficient of determination for hoarding disorder was 0.228 (adjusted R^2^ of 0.215), suggesting that the predictor constructs accounted for 22.8% of the observed variance in hoarding disorder symptoms, which can be interpreted as a moderate effect. For digital hoarding, the structural model analysis yielded a coefficient of determination of 0.376 (adjusted R^2^ of 0.363), indicating that the predictor constructs explained 37.6% of the observed variance in digital hoarding. This level of explained variance can be interpreted as representing a strong effect size.

The next step involved evaluating the model’s predictive power using PLSpredict. The results of this analysis are summarized in Table 6. The analysis indicates that the Q^2^ predict values for all indicators of the endogenous constructs were greater than zero, suggesting that the model exceeds the minimum expected reference threshold.

When comparing RMSE values, the model exhibited smaller prediction errors (i.e., smaller RMSE values) than the LM for all four hoarding disorder indicators and for all three digital hoarding indicators. These results suggest that the model demonstrates strong predictive power.

Furthermore, to confirm the model’s predictive power, the CVPAT (cross-validated predictive ability test) was performed (see Table 7). In both reports (indicator average, IA, and linear model, LM), negative average loss values were obtained for all indicators, indicating that the model exhibits lower average loss than IA and LM. Moreover, since the p-value is lower for all indicators except HD1 and LV-DE, it can be concluded that the model’s predictive capabilities are significantly superior to those of the two comparison references (Hair et al., 2022). Therefore, the CVPAT results further support the model’s strong predictive power.

Finally, although there is no consensus among researchers regarding the use of global model fit indices in PLS-SEM, several were nonetheless evaluated. The SRMR value for the model was 0.054, and the NFI was 0.802. The SRMR value lies below the recommended threshold of 0.08, indicating good absolute model fit, whereas the NFI value falls below the commonly suggested cutoff of 0.90, indicating only moderate fit. Overall, the model demonstrates acceptable global fit, particularly with respect to SRMR.

### 4.4. Robustness Analysis

To assess the model’s robustness, two additional analyses were conducted: a comparison between the second-stage model with an alternative specification, and an examination of the relationship among constructs after controlling for the explanatory variables.

Following prior recommendations, different configurations were compared (Guenther et al., 2023; Hair et al., 2022). Specifically, the final model (Figure 3) was compared with the final first-stage model (Figure 2) as suggested by Hair et al. (2024). Table 8 presents the results of the Bayesian information criterion (BIC) for both models.

The BIC value for the hoarding disorder construct (HD) was lower in Model 1 (the second-stage model) than in the alternative model. Although digital hoarding (DH) in Model 1 is not directly comparable to its counterpart in the alternative model, the BIC value for DH was clearly lower than those of its constituent dimensions in the alternative model. These results suggest that the final model exhibits superior performance relative to the alternative specification.

Given that the model follows a mediation design, and in line with the recommendations of Chua (2022), the PROCESS algorithm implemented in SmartPLS 4.0 was employed to examine relationships among constructs after controlling for explanatory variables. Three covariates were included in the analysis: age, sex, and number of devices.

**Figure 4:**
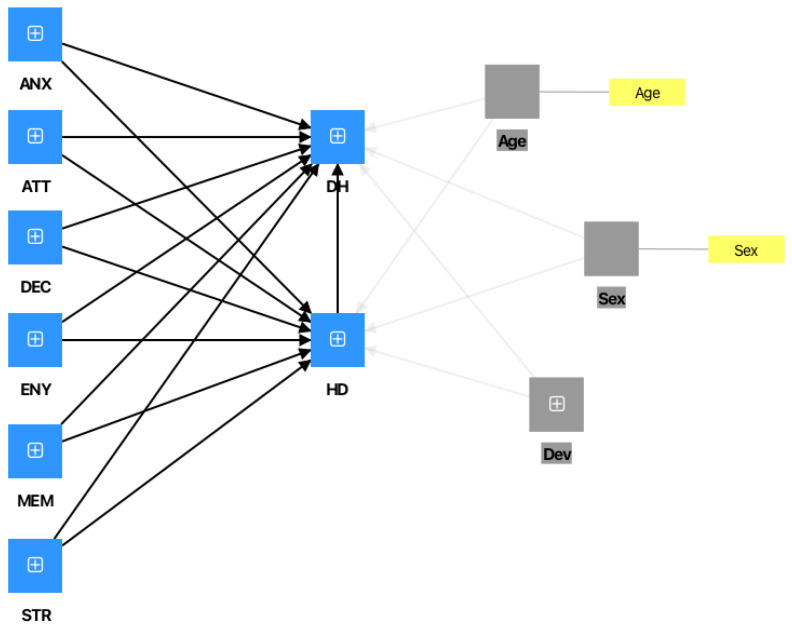
SmartPLS output for model analysis using PROCESS.

The output of the PROCESS algorithm in SmartPLS is shown in Figure 4 in the Appendix A. Table 9 presents the bootstrapping results for the control variables.

None of the control variables exerted a significant effect on the exogenous variables: hoarding disorder symptoms and digital hoarding. Moreover, a comparison between Table 9 and Figure 4 reveals that the model results with and without control variables are nearly identical. This indicates that incorporating control variables such as age, sex, and number of devices does not alter the model’s outcomes.

**Figure 5:**
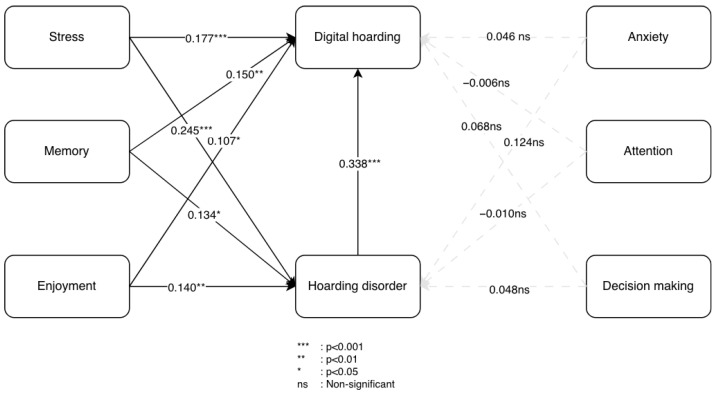
Final model of hoarding disorder and digital hoarding.

Figure 5 summarizes and illustrates the main results of the analysis. For interpretative purposes, the constructs were repositioned such that the factors significantly influencing hoarding disorder and digital hoarding appear on the left. These factors are ordered according to their level of influence on digital hoarding, from highest to lowest (i.e., stress first and enjoyment last). On the right, the three non-significant constructs are displayed. Gray dashed paths represent relationships that were not supported in the analysis.

In conclusion, the proposed model confirmed that the factors influencing hoarding disorder symptoms also affect digital hoarding. Enjoyment, memory, and stress positively and significantly predicted both hoarding disorder symptoms and digital hoarding, whereas anxiety, attention, and decision-making did not significantly influence either behavior. Among the significant exogenous factors, stress was the most influential predictor on both behaviors. Notably, while enjoyment was the second most influential factor for hoarding disorder symptoms, memory emerged as the second most influential factor for digital hoarding.

The results also confirmed that hoarding disorder symptoms mediates the relationship between digital hoarding and the factors of enjoyment, memory, and stress. The mediation model accounted for 37.6% of the variance in digital hoarding, with hoarding disorder symptoms emerging as the most influential direct predictor.

Additional tests confirmed the robustness of the model. The Q^2^ predict and CVPAT results supported its strong out-of-sample predictive power. After controlling for age, sex, and number of devices, the relationships remained stable, and none of the control variables exhibited significant effects within the model.

## 5. Discussion

The main objective of this research was to examine whether the factors traditionally associated with hoarding disorder operate similarly in digital hoarding and, additionally, to evaluate whether hoarding disorder exerts a direct influence on digital hoarding. To this end, emotional and cognitive factors, namely anxiety, stress, enjoyment, attention, memory, and decision-making, were analyzed: as predictors of both behaviors. The results partially confirm the convergence between the two forms of hoarding, revealing that enjoyment, memory, and stress positively and significantly predict both hoarding disorder and digital hoarding. In contrast, anxiety, attention, and decision-making did not exhibit significant effects on either behavior.

From an applied perspective, the effect sizes reported in Table 3 indicate that, among university students, the relationship between hoarding disorder symptoms and digital hoarding is sufficiently strong to justify integrated interventions that simultaneously address both problematic behavioral patterns. Therefore, it is very important to identify young individuals exhibiting high levels of both hoarding disorder symptoms and digital hoarding as a priority subgroup for the prevention of academic and mental health difficulties. Furthermore, the significant associations between digital hoarding, stress, and memory suggest that digital hoarding is linked to subjective stress burden and to perceived memory failures associated with cognitive overload, a higher likelihood of errors, and consequently, lower study effectiveness. Although these effect sizes are small, they support the development of interventions by health or educational institutions to help young people avoid being affected by mental health problems that ultimately impact their academic performance, as suggested by recent research on digital hoarding (Bravo-Adasme et al., 2025). For example, health and educational institutions could implement training in digital file and object organization, digital hygiene routines, and stress regulation techniques (such as mindfulness), among others.

Looking at the explained variance (R^2^ and R^2^ adj) of the model for both hoarding disorder symptoms and digital hoarding, practical conclusions can be drawn. The model explained 22.8% of the variance in hoarding disorder symptoms (R^2^ adj = 0.215, moderate effect), indicating that stress, enjoyment, and memory capture an important portion of the mechanisms underlying hoarding behavior among university students. For digital hoarding, the explained variance reached 37.6% (R^2^ adj = 0.363; strong effect), suggesting that the evaluated factors, together with the mediation of hoarding disorder symptoms, represent consistent predictors of this problematic behavior associated with information technology use. In particular, the effect size of hoarding disorder symptoms on digital hoarding (f^2^ = 0.141) implies, in practical terms, that students with hoarding symptoms have a high probability of also presenting digital hoarding symptoms. This relationship supports the need to design integrated interventions that simultaneously address both problematic behaviors by mental health and educational institutions.

Regarding anxiety, the results indicate that it does not exert a significant influence on either hoarding disorder or digital hoarding. These findings partially contradict the existing literature on HD but align with the limited empirical evidence that exists on digital hoarding. In the case of hoarding disorder, several studies have established a relationship between anxiety and this behavior (Frost et al., 2011; Timpano et al., 2013), indicating that hoarding acts as an emotional regulation mechanism that reduces anxiety associated with discarding objects (Steketee et al., 2003). However, other research has proposed that the relationship between anxiety and HD may be mediated by other factors or be less direct. With respect to the relationship with digital hoarding, prior studies suggest that anxiety may play a role in DH, particularly when individuals delete digital content, such as emails or photographs (Luxon et al., 2019; Mckellar et al., 2020). However, these findings stem primarily from qualitative or exploratory studies, which, although providing valuable information, lack robust empirical validation. One possible theoretical explanation for these results is that anxiety may exert its influence indirectly or be mediated by other factors, becoming salient only when hoarding behaviors reach clinical severity—although this interpretation remains speculative and requires further empirical examination. In the specific context of university students, it is plausible that elevated anxiety levels tend to occur during exam periods or assignments’ deadlines; however, it should be noted that such emotional responses are situational and may not translate into hoarding behaviors in non-clinical populations.

In contrast, stress produced markedly different results. Both proposed hypotheses (H2 and H8) were supported, indicating that stress significantly influences both hoarding disorder and digital hoarding. These findings are consistent with previous literature on hoarding behavior. Regarding hoarding disorder, research has demonstrated that stress and stressful life events are positively associated with hoarding severity (Frost & Hartl, 1996; Timpano et al., 2011), as hoarding may serve as a coping mechanism that provides a sense of security or control in response to such circumstances (Frost & Hartl, 1996). In the digital context, although research remains limited, the findings align with previous studies identifying stress as a key factor underlying hoarding behavior, especially when individuals experience information overload (Neave et al., 2019; Sweeten et al., 2018). The finding that stress is the most strongly associated factor for both types of hoarding is consistent with prior literature; however, several theoretical explanations may account for this pattern, which future research should seek to confirm empirically: (1) stress generally reduces individuals’ cognitive capacity to process information and make decisions about what to eliminate or retain in both physical and digital environments; (2) stress may act as an avoidance behavior, leading individuals to postpone organizing or deleting possessions, thereby exacerbating hoarding tendencies; and (3) stress may increase emotional attachment to possessions, making their disposal more difficult. It is important to clarify that these proposed mechanisms are theoretical in nature and are not directly tested in the present study. In the case of university students, multiple sources of stress, both academic and personal, may contribute to hoarding behaviors. In academic settings, the pressure to perform can lead students to accumulate study material, notes, books, and other documents they perceive as potentially useful. In the digital context, this tendency often extends to the storage of hundreds of emails from peers and professors, reports, study material, screenshots, and other digital files. Previous research has shown that digital hoarding is associated with higher levels of stress and reduced productivity (Sedera et al., 2022) and that it may also contribute to increased mental fatigue and diminished academic performance (Bravo-Adasme et al., 2025; Liu & Liu, 2025).

The results indicate that the hypothesis related to enjoyment was supported for both hoarding disorder and digital hoarding. In the case of HD, enjoyment emerged as the second most influential factor after stress, whereas in digital hoarding its effect was also significant, though it ranked as the third most influential factor. These findings are consistent with previous research on hoarding disorder, which has shown that, contrary to the common perception of as a purely negative behavior, individuals often experience positive emotions, such as enjoyment, towards their stored objects (Frost et al., 1998; Grisham & Barlow, 2005). This finding may be explained by the positive reinforcement that individuals experience when acquiring or saving possessions. Each item may evoke pleasurable sensations associated with the memories it represents, or the satisfaction derived from collecting it. Consequently, this positive emotional response may reinforce hoarding behavior. Regarding digital hoarding, studies examining enjoyment as an underlying cause remains scarce. Existing research has found that individuals who accumulate collections of digital items report experiencing enjoyment when saving and organizing such objects (Mckellar et al., 2020). In the digital domain, enjoyment may also operate as a form of positive reinforcement, particularly when a person completes a collection of digital files, revisits old photographs, or saves digital objects perceived as potentially useful in the future. Among students, in particular, enjoyment may be closely linked to academic behavior. Saving notes, course material, screenshots of class material, or books, whether in physical or digital formats, may evoke feelings of preparedness and security concerning their academic responsibilities, though this interpretation is speculative and should be treated as a hypothesis for future inquiry.

Furthermore, the results supported hypotheses H6 and H12, demonstrating that memory significantly influences both hoarding disorder and digital hoarding. In this case, memory emerged as the second most influential factor in digital hoarding, surpassing enjoyment in this specific context, and as the third most relevant factor in hoarding disorder. This finding aligns with previous research indicating that memory deficits and fear of forgetting are associated with HD (Hartl et al., 2004; Kyrios et al., 2018; Nedeljkovic et al., 2009). Regarding digital hoarding, the present study provides empirical evidence supporting the theoretical assumptions proposed to date about this relationship. The difference in influence of memory on each behavior suggests that specific dynamics within the digital context may amplify memory’s role in digital hoarding. This distinction can be explained by unique characteristics of the digital environment, particularly the fact that most digital items inherently contain have or represent information (e.g., emails, documents, screenshots, messages, and similar materials). The ease of digital storage may also reduce the need to delete, potentially enabling individuals to retain large amounts of content as a strategy—whether conscious or not—to avoid forgetting; however, this mechanism has not been directly tested in the present study and remains theoretical. Among students, this tendency may be further explained by their academic context, which requires managing large volumes of information, both physical and digital, and may thereby foster greater dependence on these materials as external memory aids.

With regard to attention, the findings indicate that it does not exert a significant influence on either hoarding disorder or digital hoarding. This contrasts with part of the HD literature, which has shown that individuals with attention deficits tend to exhibit higher levels of hoarding (Hartl et al., 2004; Tolin et al., 2011). However, most of these studies have been conducted with clinical samples, which could account for the discrepancy. Concerning attention’s role in digital hoarding, it has only been theoretically suggested that constant distraction and multitasking may affect the management of digital objects (Sweeten et al., 2018). The lack of a significant effect of attention on both types of hoarding may indicate that it operates more as a mediator than as a direct cause, or that attention primarily influences the organization of physical or digital objects rather than the quantity accumulated. Among students, this result may be attributable to the fact that this population is often characterized by attention difficulties associated with constant use of devices and information overload, though direct evidence supporting this interpretation in the context of hoarding behaviors remains limited.

Similarly to attention, our results indicate that decision-making does not significantly influence either type of hoarding. This finding contradicts a substantial body of research on HD, which has shown that individuals exhibiting hoarding behavior often experience marked impairments in decision-making (Dozier & Ayers, 2017; Kyrios et al., 2018; Tolin et al., 2012). Although several studies have confirmed this relationship, it is important to mention that several of these studies are based on clinical samples of individuals diagnosed with hoarding disorders. In the case of digital hoarding, the results are equally noteworthy, as the role of decision-making in this behavior has largely been extrapolated from the hoarding disorder literature without robust empirical validation. The absence of a significant relationship between decision-making and either form of hoarding may indicate that decision-making does not act as a direct predictor but rather interacts with other cognitive or emotional functions. Regarding university students, this lack of influence may also be related to characteristics of the sample, as young adults may not yet exhibit the full expression of hoarding-related decision-making impairments typically observed in clinical populations; however, this remains a tentative interpretation that was not directly examined in this study and should be explored in future research.

Finally, this research also demonstrated that hoarding disorder influences digital hoarding, while also extending this proposition by showing that HD significantly mediates the relationship between enjoyment, memory, and stress with digital hoarding. The mediation model accounted for 37.6% of the variance in digital hoarding, evidencing that hoarding disorder not only acts as a direct predictor but also as a mechanism through which emotional and cognitive factors indirectly shape digital hoarding behavior. These findings align with emerging research that has studied the relationship between the two behaviors and reported significant associations between them (Luxon et al., 2019; Thorpe et al., 2019). However, this study contributes to understanding the explanatory mechanisms linking both types of hoarding by demonstrating that disorder functions as a mediator. This suggests that the emotional and cognitive processes underlying hoarding disorder may transfer to the digital context. The results may be interpreted as suggesting that individuals who hoard physical objects may develop habitual patterns that extend into the digital domain, although this interpretation is theoretical and not directly tested here. Behaviors such as saving items due to stress, enjoyment, or fear of forgetting may foster similar patterns toward retaining digital materials, such as emails or photographs.

## 6. Conclusions

This research sought to answer two main questions: (1) are the factors that influence hoarding disorder the same as those that influence digital hoarding? and (2) does hoarding disorder influence digital hoarding? The results partially supported the first question, revealing that three of the six examined factors, stress, enjoyment, and memory, significantly predict both types of hoarding. Stress emerged as the most influential factor for both behaviors, confirming its central role in both HD and DH. However, the relative importance of the other significant factors differed between contexts. In the case of hoarding disorder, enjoyment ranked second, followed by memory, suggesting that positive emotions associated with acquiring and retaining physical possessions play a more prominent role than cognitive concerns about remembering information. Conversely, in the case of digital hoarding, memory emerged as the second most influential factor, while enjoyment ranked third. This shift in relative importance suggests that, within the digital context, concerns about forgetting information or losing access to valuable data outweigh the pursuit of emotional satisfaction as a motivator for hoarding. Finally, anxiety, attention, and decision-making showed no significant effects on either behavior.

Regarding the second research question, the results confirm that hoarding disorder symptoms directly influences digital hoarding and serves as a mediating mechanism linking stress, enjoyment, and memory to digital hoarding. The mediation model accounted for 37.6% of the variance in digital hoarding, identifying hoarding disorder as the factor exerting the strongest direct influence on this behavior. This finding suggests that digital hoarding does not emerge solely as a response to the characteristics of the digital environment but is shaped by behavioral patterns previously established in the physical domain.

From a theoretical perspective, this research offers important contributions to the understanding of digital hoarding. First, it provides empirical evidence identifying which key factors associated with traditional hoarding transfer to the digital context, and which do not, an area previously addressed only at the conceptual level. Second, stress emerges as the primary associated factor for both HD and DH, reinforcing the notion of hoarding as an emotional coping strategy that transcends different contexts. Third, the shift in the relative influence of enjoyment and memory across contexts suggests that the digital environment introduces specific dynamics that amplify certain factors while attenuating others. This leads to the conclusion that digital hoarding is not merely a contextual manifestation of hoarding disorder but a distinct behavior with its own defining characteristics that warrants specific characterization. Finally, this study contributes to a broader understanding of hoarding by suggesting that it should be understood as a dynamic behavior shaped by contextual factors.

From a practical perspective, this work presents important contributions. For the general population, the findings suggest that interventions aimed at reducing hoarding behaviors should focus on strategies to manage stress, regulate enjoyment, and address memory-related factors in hoarding. More specifically, cognitive-behavioral approaches that target dysfunctional beliefs about the need to retain objects—both physical and digital—alongside stress regulation techniques such as mindfulness-based interventions, may be particularly relevant given that stress emerged as the primary predictor of both behaviors. Moreover, since hoarding disorder mediates the relationship with digital hoarding, interventions could leverage the greater visibility of HD as an entry point for addressing digital hoarding. For university students, the implications are particularly relevant given the pervasive context of information overload in which they operate. Educational institutions could implement preventive programs early in university life, prioritizing the managing of academic stress, which emerged as the most influential factor in both types of hoarding. Concretely, these could include workshops on time management and academic stress reduction, digital literacy programs focused on organizational strategies for managing files, emails, and digital content, and awareness campaigns about the psychological consequences of digital hoarding. Likewise, training students in evidence-based stress reduction techniques and effective management of physical and digital possessions could help prevent the development of hoarding patterns before they consolidate.

This study has several limitations that should be acknowledged. First, the sample was composed exclusively of university students, which limits the generalizability of the findings to other populations such as older adults, clinical groups, or individuals with lower levels of digital engagement. The use of a convenience sample also introduces sampling bias that should be considered when interpreting the results. Second, all variables were measured through self-report instruments, which entails the risk of self-report bias, including social desirability effects and recall inaccuracy, potentially affecting the validity of responses. Third, the cross-sectional design of the study does not allow for causal inferences; while the structural model identifies predictive relationships, the directionality and causal nature of these associations cannot be established from the present data alone. Finally, the model does not account for potential unmeasured confounders, such as personality traits, attachment styles, prior mental health history, or cultural factors, which may influence both hoarding disorder and digital hoarding and whose inclusion could modify the observed relationships.

Finally, this research opens several avenues for future research. First, it would be valuable to replicate the proposed model in other populations, such as older adults, clinical samples, or working adults in high-demand occupational contexts, to further evaluate the generalizability of the factors studied and address the sampling limitations of the present work. Second, longitudinal studies would allow researchers to analyze the evolution of hoarding disorder and digital hoarding over time, and to establish causal relationships between the identified predictors and hoarding outcomes that the current cross-sectional design cannot support. Third, future studies could incorporate objective measures of hoarding behavior—such as digital storage audits or behavioral tasks—to complement self-report instruments and reduce self-report bias. Fourth, it would be relevant to examine the role of unmeasured variables not included in the present model, such as personality traits like perfectionism or neuroticism, attachment styles, and beliefs about possessions, which prior research suggests may be relevant to hoarding behavior. Finally, given that hoarding disorder emerged as a significant mediator of the relationship between emotional and cognitive factors and digital hoarding, future research could explore whether early intervention targeting HD can serve as a preventive mechanism for the development of digital hoarding, particularly in high-risk populations such as university students.

## Figures and Tables

**Table 1:** Items by construct and source reference.

Construct	Dimension	Number of Items	Reference
Attention		4	Nedeljkovic and Kyrios (2007)
Memory		15	
Decision-making		5	
Enjoyment		3	Sun and Zhang (2006)
Anxiety		7	Antony et al. (1998)
Stress		10	Cohen and Williamson (1988)
Hoarding disorder		5	Tolin et al. (2010)
Digital Hoarding	Difficulty discarding	7	Adapted from Frost et al. (2004)
	Clutter	9	
	Acquisition	7	

**Table 2:** Descriptive statistics for the sample.

	Sex	N	Mean	Median	SD	Minimum	Maximum
Age	Female	187	20.52	20.00	2.466	18	43
	Male	152	20.38	20.00	2.671	18	44
	Prefer not to say	5	19.00	19.00	1.225	18	21
Devices	Female	187	2.47	2.00	0.735	0	5
	Male	152	2.66	3.00	0.805	1	6
	Prefer not to say	5	2.40	2.00	0.894	2	4

**Table 3:** Results of structural model analysis for the conceptual model’s endogenous constructs.

Hypothesis	IV	DV	VIF	Path	T Statistics	*p* Values	Conclusion	f^2^	Effect Size
H1	ANX	HD	1.780	0.124	1.864	0.062	Rejected	0.011	NA
H7		DH	1.800	0.046	0.731	0.465	Rejected	0.002	NA
H4	ATT	HD	1.408	−0.010	0.170	0.865	Rejected	0.000	NA
H10		DH	1.408	−0.006	0.110	0.912	Rejected	0.000	NA
H5	DEC	HD	1.568	0.048	0.798	0.425	Rejected	0.002	NA
H11		DH	1.571	0.068	1.291	0.197	Rejected	0.005	NA
H3	ENY	HD	1.023	0.140	2.819	0.005	Supported	0.025	Small
H9		DH	1.048	0.107	2.053	0.040	Supported	0.017	Small
H6	MEM	HD	1.429	0.134	2.340	0.019	Supported	0.016	Small
H12		DH	1.453	0.150	2.839	0.005	Supported	0.025	Small
H2	STR	HD	2.113	0.245	3.846	0.000	Supported	0.037	Small
H8		DH	2.190	0.177	2.769	0.006	Supported	0.023	Small
H13	HD	DH	1.296	0.338	6.085	0.000	Supported	0.141	Large

IV: Independent variable; DV: Dependent variable; f^2^: Effect size.

**Table 4:** Total and indirect effects of the exogenous constructs on digital hoarding mediated by hoarding disorder symptoms.

	Indirect Effect			Total Effect		
	Path	T Statistics	*p* Values	Path	T Statistics	*p* Values
ANX → DH	0.042	1.756	0.079	0.088	1.354	0.176
ATT → DH	−0.003	0.166	0.868	−0.009	0.180	0.857
DEC → DH	0.016	0.772	0.440	0.084	1.562	0.118
ENY → DH	0.047	2.459	0.014	0.154	3.094	0.002
MEM → DH	0.045	2.144	0.032	0.195	3.626	0.000
STR → DH	0.083	3.231	0.001	0.260	4.180	0.000

**Table 5:** Coefficient of determination.

	R^2^	R^2^ Adj	Interpretation
HD	0.228	0.215	Moderate
DH	0.376	0.363	Strong

**Table 6:** PLSpredict results report.

LV Predict	Q^2^ Predict	MV Predict	Q^2^ Predict	PLS-SEM_RMSE	PLS-SEM_MAE	LM_RMSE	LM_MAE
HD	0.194	HD1	0.058	2.082	1.671	2.250	1.787
		HD3	0.096	1.868	1.498	1.996	1.569
		HD4	0.165	1.835	1.467	1.936	1.531
		HD5	0.166	1.887	1.521	1.986	1.574
DH	0.251	LV–AD	0.210	0.891	0.716	0.936	0.736
		LV–DD	0.158	0.921	0.728	0.982	0.770
		LV–DE	0.182	0.906	0.694	0.935	0.719

**Table 7:** CVPAT test results.

CVPAT MV	Indicator Average				Linear Model			
	PLS Loss	IA Loss	Average Loss Difference	*p* Value	PLS Loss	LM Loss	Average Loss Difference	*p* Value
HD1	4.334	4.603	−0.269	0.132	4.334	5.063	−0.729	0.000
HD3	3.488	3.857	−0.369	0.013	3.488	3.982	−0.494	0.002
HD4	3.369	4.033	−0.664	0.001	3.369	3.748	−0.379	0.007
HD5	3.562	4.273	−0.711	0.001	3.562	3.943	−0.381	0.013
LV–AC	0.795	1.006	−0.212	0.000	0.795	0.876	−0.081	0.046
LV–DD	0.849	1.007	−0.159	0.004	0.849	0.964	−0.115	0.001
LV–DE	0.821	1.005	−0.183	0.000	0.821	0.874	−0.053	0.134

**Table 8:** BIC values for model comparison.

Model 1 (Second-Stage)	BIC	Model 2 (First-Stage)	BIC
DH	−116.398	AD	−75.043
		DD	−59.777
		DE	−90.174
HD	−49.302	HD	−46.000

**Table 9:** Bootstrapping results from PROCESS for control variables.

	Path	T Statistics	*p* Values
Age ← DH	−0.005	0.346	0.729
Age ← HD	−0.008	0.446	0.656
Dev ← DH	0.095	1.570	0.116
Dev ← HD	0.023	0.337	0.736
Sex ← DH	−0.011	0.123	0.902
Sex ← HD	0.075	0.632	0.527

## Data Availability

The data that support the findings of this study are available on request from the corresponding author.

## Appendix A

**Table A1:** Factor loadings and VIF values of items in the first-order model.

	ANX	ATT	DEC	AC	DD	DE	ENY	HD	MEM	STR	VIF
ANX1	0.729										1.855
ANX3	0.789										1.983
ANX4	0.730										1.707
ANX5	0.815										2.218
ANX6	0.854										2.649
ANX7	0.799										2.095
ATT1		0.819									2.836
ATT2		0.860									3.291
ATT3		0.892									2.771
ATT4		0.893									2.396
DEC_1			0.873								2.945
DEC_2			0.886								3.158
DEC_3			0.763								1.771
DEC_4			0.804								2.086
DEC_5			0.819								1.964
DH_AC1				0.704							1.493
DH_AC2				0.737							1.527
DH_AC3				0.709							1.393
DH_AC4				0.812							2.131
DH_AC5				0.727							1.647
DH_DD4					0.817						1.802
DH_DD5					0.755						1.532
DH_DD6					0.753						1.407
DH_DD7					0.796						1.637
DH_DE1						0.777					1.848
DH_DE2						0.733					1.872
DH_DE4						0.773					1.924
DH_DE5						0.736					1.666
DH_DE6						0.709					1.586
DH_DE8						0.775					2.030
DH_DE9						0.755					1.776
ENY1							0.879				2.516
ENY2							0.869				2.439
ENY3							0.914				2.059
HD1								0.707			1.519
HD2								0.678			1.509
HD3								0.731			1.578
HD4								0.849			2.252
HD5								0.853			2.293
MEM1									0.824		3.847
MEM10									0.804		2.649
MEM11									0.728		2.050
MEM13									0.797		2.736
MEM14									0.769		2.388
MEM2									0.857		4.550
MEM3									0.824		3.404
MEM4									0.759		2.249
MEM5									0.717		2.127
MEM6									0.817		2.796
MEM7									0.838		3.520
MEM8									0.865		3.967
MEM9									0.767		2.361
STR1										0.729	1.744
STR10										0.785	1.905
STR2										0.763	1.597
STR3										0.751	1.690
STR6										0.679	1.456
STR9										0.685	1.627

**Table A2:** Reliability, convergent validity, and discriminant validity values of the first-order model.

	CA	CR (rho_c)	AVE	AC	ANX	ATT	DD	DE	DEC	ENY	HD	MEM
AC	0.791	0.857	0.546									
ANX	0.877	0.907	0.620	0.382								
ATT	0.893	0.923	0.751	0.276	0.387							
DD	0.787	0.862	0.610	0.824	0.308	0.211						
DE	0.872	0.901	0.565	0.641	0.403	0.262	0.748					
DEC	0.886	0.917	0.689	0.297	0.472	0.467	0.366	0.378				
ENY	0.870	0.917	0.787	0.374	0.097	0.074	0.205	0.092	0.086			
HD	0.824	0.876	0.588	0.472	0.392	0.251	0.519	0.558	0.356	0.221		
MEM	0.952	0.958	0.638	0.373	0.363	0.466	0.362	0.385	0.477	0.144	0.340	
STR	0.828	0.874	0.537	0.465	0.756	0.500	0.461	0.484	0.612	0.116	0.470	0.482

CA: Cronbach’s alpha; CR: Composite reliability; AVE: Average variance extracted.

**Table A3:** Reliability, convergent validity, and discriminant validity values of the second-order model.

	CA	CR (rho_c)	AVE	ANX	ATT	DEC	DH	ENY	HD	MEM
ANX	0.877	0.907	0.620							
ATT	0.893	0.923	0.751	0.387						
DEC	0.886	0.917	0.689	0.472	0.467					
DH	0.821	0.893	0.737	0.425	0.291	0.406				
ENY	0.870	0.917	0.787	0.097	0.074	0.086	0.249			
HD	0.808	0.875	0.638	0.405	0.246	0.338	0.622	0.231		
MEM	0.952	0.958	0.638	0.363	0.466	0.477	0.436	0.144	0.353	
STR	0.814	0.869	0.571	0.759	0.488	0.602	0.542	0.119	0.487	0.468

CA: Cronbach’s alpha; CR: Composite reliability; AVE: Average variance extracted.

**Table A4:** Factor loadings and VIF values of items in the second-order model.

	ANX	ATT	DEC	ENY	HD	DH	MEM	STR	VIF
ANX1	0.727								1.855
ANX3	0.787								1.983
ANX4	0.730								1.707
ANX5	0.815								2.218
ANX6	0.854								2.649
ANX7	0.802								2.095
ATT1		0.821							2.836
ATT2		0.859							3.291
ATT3		0.890							2.771
ATT4		0.894							2.396
DEC_1			0.874						2.945
DEC_2			0.887						3.158
DEC_3			0.766						1.771
DEC_4			0.801						2.086
DEC_5			0.816						1.964
ENY1				0.872					2.516
ENY2				0.875					2.439
ENY3				0.914					2.059
HD1					0.718				1.490
HD3					0.717				1.390
HD4					0.864				2.172
HD5					0.881				2.293
LV–AC						0.844			1.831
LV–DD						0.881			2.152
LV–DE						0.849			1.721
MEM1							0.823		3.847
MEM10							0.804		2.649
MEM11							0.728		2.050
MEM13							0.798		2.736
MEM14							0.770		2.388
MEM2							0.857		4.550
MEM3							0.823		3.404
MEM4							0.760		2.249
MEM5							0.718		2.127
MEM6							0.817		2.796
MEM7							0.836		3.520
MEM8							0.864		3.967
MEM9							0.766		2.361
STR1								0.741	1.728
STR10								0.793	1.804
STR2								0.766	1.499
STR3								0.764	1.680
STR9								0.711	1.627
